# Central Texas Medical Society—Proceedings of the Last Meeting

**Published:** 1889-08

**Authors:** W. O. Wilkes

**Affiliations:** Secretary; Waco, Texas


### SOCIETY NOTES.

### CENTRAL TEXAS MEDICAL SOCIETY.

WACO, TEXAS, July 26, 1889.

*Editor Daniel's Texas Medical Journal:*

The twelfth quarterly meeting of the Central Texas Medical Association, which convened in this city Tuesday, July 9, was equal to any of the preceding meetings, in point of interest, and as well attended as could be expected at this season of the year.

The first thing on the programme, after the preliminary business, was the reading of a paper on

PUERPERAL ECLAMPSIA, by Dr. J. C. Shaw, of Stranger. The doctor's paper was well prepared, and showed a depth of research which drew forth commendations from every one. The doctor's great *sine qua non* in the treatment is phlebotomy.

The discussion which followed the reading of the paper, was mainly confined to the methods of treatment used by the members, and prophylaxis. Some used veratrum and morphia, some chloral, some chloroform, some bromides, and some all of these remedies; but the majority seemed to favor blood-letting. Some of the members made a distinction between cases due to albuminuria and those due to pressure of blood upon the nervous centers, and thought they should be treated differently. The discussion was general, and enjoyed by all.

Dr. B. C. Brown next presented a case of a young man who was kicked on the forehead by a horse ten years ago. Seemed to be a slight depression at the point. Fifteen months ago patient began to have convulsions, which bromides controlled for only a short time.

Dr. E. D. Taylor presented a case of what he called paralysis agitans, which was also discussed by the Association.

Dr. W. R. Blailock, of McGregor, read a paper on

THE USE OF THE MICROSCOPE IN THE PRACTICE OF MEDICINE.

The paper was well received, but not discussed to any great extent. The doctor said: "The day is soon coming when the physician who does not use the microscope will fall behind in the race." He laid particular stress on its use as an aid to diagnosis.

The Secretary next read a paper furnished by Dr. C. F. Paine, of Comanche, on

ANTIPYRETICS.

The paper received as much praise as any that has ever been read in this Association. The discussion was very long, and one of the most interesting it has ever been the pleasure of the writer to listen to. The members were about equally divided as to the relative value of antipyrin and antifebrin, some favoring the one, some the other, while other antipyretics sank into insignificance beside these two. A very small number, about three members, did not favor the use of antipyretics at all; and quite a number opposed their use in pneumonia. There was at least one advocate of the theory of the conservatism of fever.

A letter was read from Dr. A. P. Brown, of Fort Worth, asking the co-operation of the association in preparing a creditable display of the medicinal minerals and plants of Texas, at the next karporama.

A letter was read from Dr. T. O. Hynes, of Brenham, describing the cases of cerebro-spinal meningitio, which came under his observation during the recent epidemics in his city.

THE EFFECT OF TOBACCO ON THE EYE-SIGHT

a paper furnished by Prof. F. B. Tiffany of Kansas City, Mo., was read. The paper was well received and discussed by the members. The President stated that the paper was a perfect description of his condition ten or twelve years ago, when he was almost blind from the use of tobacco, and he was very glad that the attention of the profession had been called to this subject by the very able paper of Prof. Tiffany.

Dr. A. H. Snead, of Waco, read an interesting paper on

FRACTURES OF THE FEMUR, and exhibited the apparatus, with which he treats fractures of the shaft of the bone. He does not approve of immediately placing the limb in a plaster splint in the majority of cases of fractures of the fennur. The doctor's apparatus is a slight modification of the Hodgen splint.

The paper was well discussed, the majority of the members favoring the placing of a plaster splint immediately upon setting the bone.

After an hour pleasantly passed in the air discussion of interesting cases, the association adjourned to meet again Tuesday, October 8th, 1889.

The programme for the next meeting is as follows: "Obesity," by Dr. M. O. Wright; "Diphtheria," by Dr. O. I. Halbert; "Pyrexia," by Dr. W. H. Wilkes; "Injuries Incident to Parturition," by Dr. P. M. Kuykendall; "Medical Experts," by Dr. H. L. Parsons. W. O. WILKES, M. D., Secretary.

---

*Ed. Daniel's Texas Medical Journal:*

DEAR DOCTOR:—The Thirteent Quarterly Session of the Central Texas Medical Association will convene in Waco, Tuesday, October 8th, 1889. Papers will be read as follows:

"Obesity," by Dr. M. O. Wright. "Diptheria," by Dr. O. I. Halbert. "Injuries Incident to Parturition—Their Consequences and Treatment," by Dr. P. M. Kuykenndall. "Medical Experts—Their Rights and Obligatiods," by Dr. H. L. Parsons. "Pyrexia," by Dr. W. H. Wilkes.

This notice is to give ample time for reading up these subjects, so that you may come to the meeting "Loaded for B'ar."

Volunteer papers always acceptable; and their titles should be sent to the Secretary as early as possible.

H. C. GHENT, Pres. W. O. WILKES, Secy.

